# Estimating ventilation rates in rooms with varying occupancy levels: Relevance for reducing transmission risk of airborne pathogens

**DOI:** 10.1371/journal.pone.0253096

**Published:** 2021-06-24

**Authors:** Arminder K. Deol, Danny Scarponi, Peter Beckwith, Tom A. Yates, Aaron S. Karat, Ada W. C. Yan, Kathy S. Baisley, Alison D. Grant, Richard G. White, Nicky McCreesh

**Affiliations:** 1 Department of Infectious Disease Epidemiology, TB Centre, The London School of Hygiene & Tropical Medicine, London, United Kingdom; 2 Department of Medicine, University of Cape Town, Cape Town, South Africa; 3 The Institute for Global Health and Development, Queen Margaret University, Edinburgh, United Kingdom; 4 Department of Infectious Disease, Faculty of Medicine, Imperial College London, London, United Kingdom; 5 Section of Immunology of Infection, Department of Infectious Disease, Imperial College London, London, United Kingdom; 6 Department of Infectious Disease Epidemiology, The London School of Hygiene & Tropical Medicine, London, United Kingdom; 7 Africa Health Research Institute, School of Laboratory Medicine & Medical Sciences, College of Health Sciences, University of KwaZulu-Natal, Durban, South Africa; 8 School of Public Health, University of the Witwatersrand, Johannesburg, South Africa; University of Surrey, School of Veterinary Medicine, UNITED KINGDOM

## Abstract

**Background:**

In light of the role that airborne transmission plays in the spread of SARS-CoV-2, as well as the ongoing high global mortality from well-known airborne diseases such as tuberculosis and measles, there is an urgent need for practical ways of identifying congregate spaces where low ventilation levels contribute to high transmission risk. Poorly ventilated clinic spaces in particular may be high risk, due to the presence of both infectious and susceptible people. While relatively simple approaches to estimating ventilation rates exist, the approaches most frequently used in epidemiology cannot be used where occupancy varies, and so cannot be reliably applied in many of the types of spaces where they are most needed.

**Methods:**

The aim of this study was to demonstrate the use of a non-steady state method to estimate the absolute ventilation rate, which can be applied in rooms where occupancy levels vary. We used data from a room in a primary healthcare clinic in a high TB and HIV prevalence setting, comprising indoor and outdoor carbon dioxide measurements and head counts (by age), taken over time. Two approaches were compared: approach 1 using a simple linear regression model and approach 2 using an ordinary differential equation model.

**Results:**

The absolute ventilation rate, Q, using approach 1 was 2407 l/s [95% CI: 1632–3181] and Q from approach 2 was 2743 l/s [95% CI: 2139–4429].

**Conclusions:**

We demonstrate two methods that can be used to estimate ventilation rate in busy congregate settings, such as clinic waiting rooms. Both approaches produced comparable results, however the simple linear regression method has the advantage of not requiring room volume measurements. These methods can be used to identify poorly-ventilated spaces, allowing measures to be taken to reduce the airborne transmission of pathogens such as *Mycobacterium tuberculosis*, measles, and SARS-CoV-2.

## Introduction

At the time of writing, over two million people have died from COVID-19 and there have been close to 100 million cases reported worldwide [1]. The world has taken unprecedented measures to control its spread. The role of droplet infection in transmission was established very early in the pandemic, and current World Health Organization (WHO) COVID-19 control guidelines list a number of measures aimed at reducing or preventing droplet and fomite transmission, such as maintaining at least a 1 metre distance from others and regular hand washing [2]. However, it is now recognised that airborne transmission also plays a role in the spread of SARS-CoV-2 necessitating a range of additional control measures [3–5].

Well-known and long established airborne infectious diseases continue to cause large numbers of deaths, with tuberculosis (TB) and measles claiming an approximately 1.4 million and over 200,000 lives in 2019 respectively [6, 7]. Work on TB and other airborne infectious diseases highlight the crucial role that ventilation levels play in transmission risk, especially in low- and middle-income settings with high TB and HIV prevalence [8–10], and studies have shown that transmission could be reduced if facilities were better ventilated, particularly in key buildings such as clinics [11–14]. To help prevent transmission of pathogens by the airborne route, WHO has previously recommended natural ventilation of at least 60 ls^-1^/patient for general outpatient departments and wards [15]. To help identify inadequately ventilated spaces, however, it is necessary to be able to estimate levels of ventilation.

Two methods are commonly used in epidemiological research to estimate ventilation rates in indoor spaces. The first method is to estimate ventilation rates using carbon dioxide (CO_2_) release experiments; that is, releasing CO_2_ into an empty room and measuring the rate of CO_2_ decay. These data can then be used to estimate ventilation rates [16]. However, this method may not be feasible in a clinic setting, a) because the space must be empty of people (not always possible) and b) because in large spaces that cannot easily be made airtight, it may not be possible to achieve the peak CO_2_ levels needed to perform accurate experiments.

The second method to characterise ventilation and indoor air quality in a room is using the steady-state methods demonstrated in Persily and de Jonge [17]. This is a simple and more practical approach to determining ventilation rates. The method only requires measurement/estimation of the steady-state outdoor and indoor CO_2_ levels and occupancy, and makes assumptions about the CO_2_ generation rate per person, which the authors defined for a range of ages and levels of physical activity. Though this approach can be easily implemented in a clinical setting, the steady-state method may not accurately estimate the true ventilation rate, as the number of room occupants and CO_2_ concentrations are unlikely to be constant.

Ventilation measurements obtained from these, or related methods, can then be used to estimate the potential risk of infection in an indoor space. The Wells-Riley model [18, 19] (Eq 1) is an example of a method that can be used, under steady-state conditions, to estimate the probability of infection in a susceptible individual (*P*). The input parameters are: the number of infectious individuals present (*I*), the number of infectious doses (‘quanta’) produced by each infectious individual per unit time (*q*) [20], the volume of air inhaled by susceptible people per unit time (*p*), the absolute ventilation rate (*Q*), and time (*t*). Usually, I, p and q have to be assumed.

$$P=1-\exp\;\left(-\frac{Ipqt}{Q}\right)$$ (1)

Rudnick and Milton [19] adapted Eq 1 to allow for non-steady state conditions (Eq 2). Here, n is the number of people in the ventilated space and $\bar{f}$ is the average fraction of indoor air that is exhaled breath:

$$P=1-exp\left(-\frac{\bar{f}Iqt}{n}\right)$$ (2)

where $\bar{f}$ can be calculated from:

$$\bar{f}=\frac{C_{in}-C_{out}}{c_{a}}$$ (3)

where C_in_ is the volume fraction of CO_2_ in indoor air, C_out_ is the volume fraction of CO_2_ in outdoor air, and C_a_ is the volume fraction of CO_2_ added to exhaled breath [19]. This approach has been widely adopted but does not permit disaggregation of the contributions that overcrowding versus poor ventilation make to transmission risk.

In this paper, we demonstrate the application of a simple non-steady state method to calculate absolute ventilation rates in a busy clinic waiting area with fluctuating occupancy. This method is suitable for routine use in such spaces, during operational hours, and requires no additional equipment beyond the CO_2_ dataloggers typically used in such research in epidemiology.

## Methods

### Data

The methods were applied using data from the *Umoya omuhle* project [21], a large multi-disciplinary research project that aims to generate novel interventions for tuberculosis infection prevention and control (IPC) in primary healthcare clinics in Western Cape and KwaZulu-Natal, two provinces in South Africa. As part of this project, ventilation measurements were undertaken in clinical spaces in ten primary healthcare clinics, using a combination of both CO_2_ release experiments and paired indoor and outdoor CO_2_ measurements. Here, we focus on one naturally ventilated clinic waiting room.

Datalogging Indoor Air Quality Meters, model 800050 (Sper Scientific, Scottsdale, Arizona; accuracy +/- 75 ppm) were used to measure CO_2_ levels. CO_2_ measurements were taken at three central locations within the room, with one concurrent measurement taken immediately outside of the space to measure CO_2_ in the replacement air. Sets of measurements were taken approximately every 20 minutes with headcounts of room occupants (by age category) collected concurrently by research staff. Room dimensions were measured using a laser distance meter (Bosch PLR 40R, Robert Bosch GmbH Gerlingen, Germany, accuracy +/- 2.0mm), and used to estimate room volumes. All data were entered in Microsoft Excel and data analyses were carried out using R version 3.6.0 [22].

### Models

#### Steady state approach

The methods applied in this study expand upon the model used by Persily and de Jonge [17]. In the original study, the authors described the relationship between steady-state CO_2_ concentration and ventilation rate as follows:

$$Q=\frac{G}{C_{in,ss}-C_{out}}$$ (4)

Where G is the CO_2_ generation rate per person (taken from [17]), C_out_ is the outdoor concentration of CO_2_, and Q and C_in,ss_ are the steady-state ventilation rate per person and indoor CO_2_ concentration, respectively (Table 1). This method does not allow for non-steady state CO_2_ or number of occupants, but is rather a ‘snapshot’ of the situation, and will be inaccurate if occupancy or ventilation levels vary.

**Table 1:** Definitions of parameters.

Parameter	Definition	Units
m_in_	(C_in_ /1x10^6^)V = volume of CO_2_ in the room	l
C_in_	concentration of CO_2_ in the room	ppm
C_out_	concentration of outdoor CO_2_	ppm
V	room volume	l
Q	Ventilation rate	ls^-1^
n	Number of individuals (occupancy)	-
G	Total CO_2_ generation rate = (n_age_1_G_age_1_ + n_age_2_G_age_2_… n_age_i_ G_age_i_)	ls^-1^
t	Time elapsed from start of data collection	s

#### Non-steady state approach

The method used by Persily and de Jonge [17] was adapted to allow for changing concentrations of indoor CO_2_ and number of occupants. Two approaches were investigated: approach 1, using simple linear regression, and approach 2, which calculated the rate of change in CO_2_ concentration accounting for the number of individuals at each elapsed time point, t, using ordinary differential equations.

For both approaches, the mean indoor CO_2_ concentration was calculated at each time point across the three monitors. The total CO_2_ generation rate (G) at each time point was estimated by multiplying the number of individuals in each age group in the room at that time point by the corresponding G for those individuals, using the reference values provided by Persily and de Jonge [17]. Both approaches assumed a well-mixed air space. For approach 2, the differential equation (Eq 9) was simple enough to be solved analytically. The formula for the indoor CO_2_ concentration was expressed in terms of the integral of the outdoor CO_2_ concentration and the generation rate (G) over time. Since such quantities were known at the 10 points in time when measurement were taken, the integral was approximated using the trapezoidal rule between those points. For the sensitivity analysis, we evaluated the effects on the estimated ventilation rates (using both approaches) of assuming different rates of occupant metabolic activity.

To determine the best fitting model (between approach 1 and approach 2), the sum of squares due to regression (SSR) was used, where the smallest value of SSR represented the best fitting model to the clinic data.

*Approach 1*. *Simple linear regression*. This was a direct adaptation from Eq (4). We fit a simple linear regression model for the relationship between the difference in CO_2_ concentration (Cin−C_out_) at each time point (Table 1) and the total CO_2_ generation rate at each time point (n(t)G, which is given by = n_age_1_G_age_1_ + n_age_2_G_age_2_… n_age_i_ G_age_i_), where the slope of the line provided Q. Note that to ensure that a total generation rate of zero corresponded to no difference in CO_2_ concentration, we constrained linear the y- intercept to be zero.

*Approach 2*. *Ordinary differential equation for non-steady state model*. The rate of change of CO_2_ in the room was calculated by:

$$\frac{dm_{in}}{dt}=C_{out}Q-C_{in}Q+n(t)G$$ (5)

where the term n(t)G = n_age_1_G_age_1_ + n_age_2_G_age_2_… n_age_i_ G_age_i_ represents the individuals contributing to exhaled air.

Dividing both sides of the equation by V and substituting C_in_ = m_in_ / V (from Table 1), we get:

$$\frac{dC_{in}}{dt}=\frac{(C_{out}-C_{in})Q+n(t)G}{V}.$$ (6)

Eq (5) is a linear differential equation of first order, which can be solved analytically using an integrating factor. Bringing all terms in *C*_*in*_ to the left, we get the equation in its standard form

$$\frac{dC_{in}}{dt}+\frac{Q}{V}\;C_{in}=\frac{C_{out}Q+n(t)G}{V}.$$ (7)

The integrating factor is then

$$u(t)=\exp\;\left(\int \frac{Q}{V}dt\right)=\exp\left(\frac{Q}{V}\text{t}\right)$$ (8)

and the solution of Eq (6) is

$$C_{in}(t)=u{\left(t\right)}^{-1}\left(C_{in}(0)+\int_{0}^{t}u(t^{\prime })\left(\frac{C_{out}(t^{\prime })Q+n(t^{\prime })G}{V}\right)dt^{\prime }\right).$$

Substituting (8) we obtain

Cin(t)=exp(−QVt)(Cin(0)+∫0texp(QVt')(Cout(t′)Q+n(t′)GV)dt′). (9)

For any given value of Q, the integrand exp(QVt')(Cout(t′)Q+n(t′)GV) in (9) was known at the 10 points in time in Table 2: the integral in (9) (and therefore the value of *C*_*in*_(*t*) was then approximated using the trapezoidal rule between those 10 points in time.

**Table 2:** Carbon dioxide (CO_2_) measurements taken immediately outside the room, from three CO_2_ meters at central locations within the room, and concurrent headcounts of room occupants.

Time	Time elapsed (s)	Outdoor CO_2_ conc. (ppm*)	Indoor1	Indoor2	Indoor3	Older children and adults	Children (1–5 years)	Infants (<1 years)	Total occupa-ncy
			CO_2_ conc. (ppm*)	CO_2_ conc. (ppm*)	CO_2_ conc. (ppm*)				
						(>5 years)			
9:40	0	398	408	425	505	37	6	1	44
10:00	1200	373	422	428	483	32	6	2	40
10:21	2460	403	438	449	464	26	7	1	34
10:40	3600	403	416	436	456	29	6	2	37
11:03	4980	401	401	420	432	19	2	1	22
11:25	6300	411	401	399	403	7	1	1	9
11:50	7800	406	400	397	396	6	0	0	6
12:09	8940	409	402	402	402	4	0	0	4
12:34	10440	398	392	396	398	1	0	0	1
12:50	11400	399	400	400	401	2	1	0	3

*ppm = parts per million.

The model was fitted to the ventilation data collected from the clinic room and the best fitting value of Q was determined by minimising the residual sum of squares. The 95% confidence interval was calculated through bootstrap resampling where 1000 iterations were carried out to develop a marginal range of values of Q in order to derive 2.5% and 97.5% percentiles.

### Ethics approval and consent to participate

This study received ethical approval from the Biomedical Research Ethics Committee of the University of KwaZulu-Natal (ref. BE082/18), the Human Research Ethics Committee of the Faculty of Health Sciences of the University of Cape Town (ref. 165/2018), the Research Ethics Committee of Queen Margaret University (ref. REP 0233), and the Observational/Interventions Research Ethics Committee of the London School of Hygiene & Tropical Medicine (ref. 14872).

## Results

Head counts for the clinic waiting room showed a higher level of occupancy in the early part of the morning, falling over the period of measurement (Table 2). The average outdoor CO_2_ concentration was 400 ppm and the average indoor CO_2_ concentration (across three monitors and all time points) was 419 ppm. Mean room occupancy throughout the data collection period (of 3 hours 10 mins) was 20 individuals. The room volume was measured to be 135,363 litres (Table 3).

**Table 3:** Results of approach 1 (linear regression) and approach 2 (model fit) for estimating the absolute ventilation rate (Q) in the clinic waiting room.

	Approach 1	Approach 2
**Room use**	Waiting area	
**Volume of space (l)**	135363	
**Duration of measurement (s)**	11400	
**SSR**	2 x 10^−9^	1.7 x 10^−9^
**Absolute ventilation rate, Q (95% CI, ls**^**-1**^**)**	2407 (1632–3181)	2743 (2139–4429)

SSR: sum of squares due to regression; CI: confidence interval.

**Fig 1:**
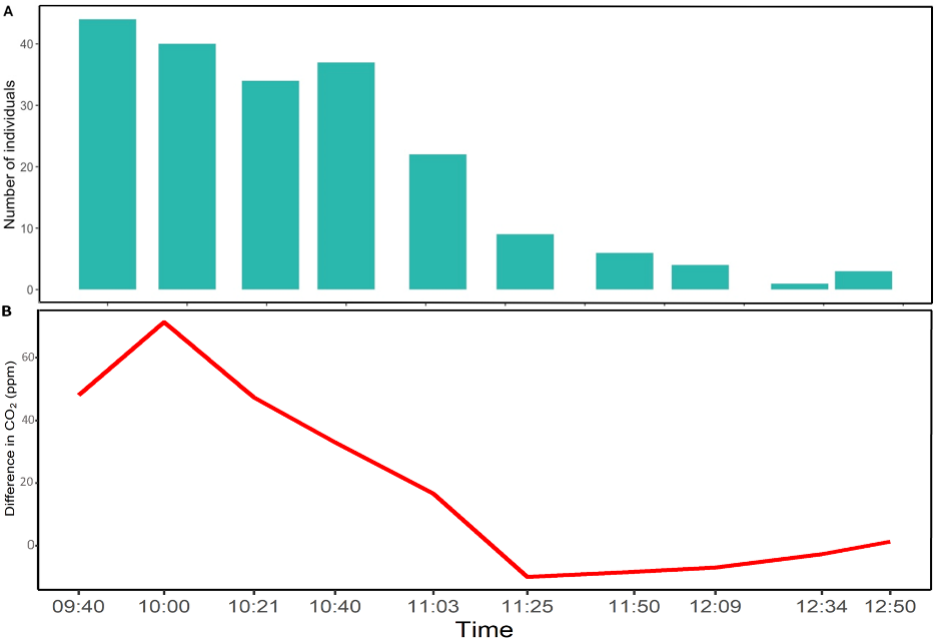
Number of individuals (A) and the difference in CO_2_ concentration (ppm) between the indoor and outdoor meters (B) over time.

The concentration of CO2 in the air varied with the number of people in the clinic room (Fig 1), as would be expected.

In the primary analysis, the level of physical activity was assumed to be 1.2 metabolic equivalents (MET), assuming occupants were sitting quietly. The corresponding CO_2_ generation rates (G) were obtained from Persily and de Jonge [17]: the <1 year old age group (G = 0.00105 ls^-1^), 1 to 5 year olds (G = 0.001975 ls^-1^) and those in all age categories above this group (G = 0.00377 ls^-1^ [17]; which is the mean of G provided for all older age brackets).

**Fig 2:**
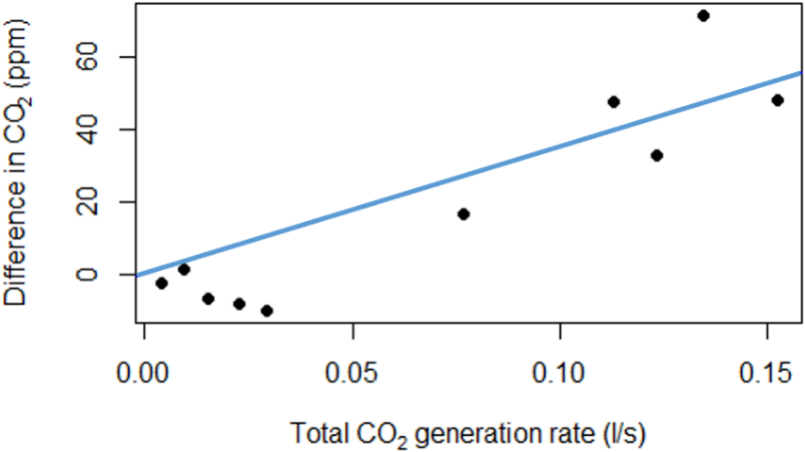
Difference between indoor and outdoor CO_2_ readings (ppm = parts per million) against the total CO_2_ generation rate at each time point (ls^-1^). Line represents the best fit by linear regression with y-intercept constrained to be zero.

**Fig 3:**
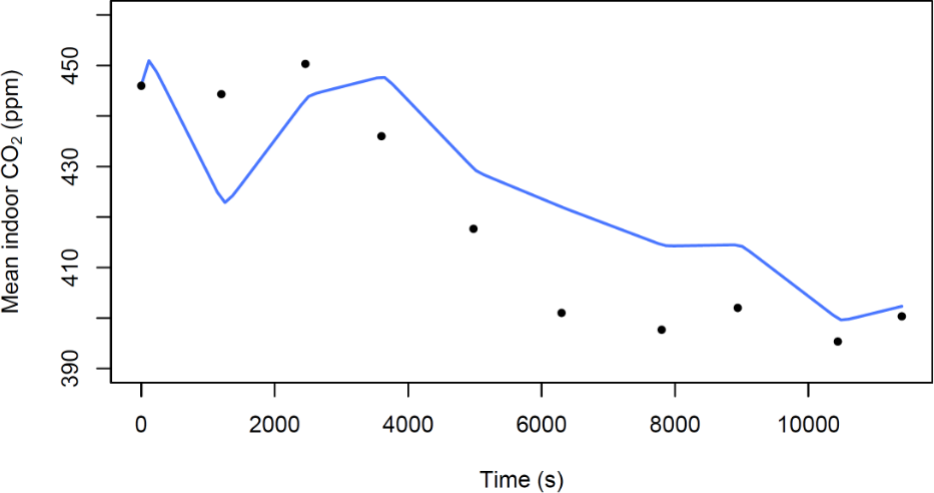
Mean concentration of indoor CO_2_ (ppm = parts per million) vs time elapsed since start of data collection (s). The line represents the fitted model from approach 2 and the black dots are the data points.

Both approaches showed comparable results though, using the SSR, approach 2 was found to be the best fitting model (Figs 2 and 3 for model fits and Table 3). The absolute ventilation rate was quantified as 2407 ls^-1^ (95% CI: 1632–3181) and 2743 ls^-1^ (95% CI: 2139–4429) for approach 1 and approach 2, respectively (Table 3).

### Sensitivity analyses

We compared our original estimate, which assumed a metabolic activity of 1.2 MET, with estimates assuming 1.0, 1.4, and 1.6 MET (Table 4). For example, 1.0–1.3 MET represent states such as lying down, sitting quietly (such as when reading or writing), or standing still; 1.5 MET is seen when sitting whilst carrying out light tasks, such as office work; and 3.0 MET is seen in individuals carrying out light standing tasks, such as filing [17]. Note, certain disease states would be expected to increase the metabolic rate.

**Table 4:** Carbon dioxide generation rate (ls^-1^) in each age group for each level of metabolic activity (MET [17]).

Metabolic activity (MET)		1.0	1.2	1.4	1.6
**CO**_**2**_ **generation rate in each age group (ls**^**-1**^**)**	<1 year olds	0.0009	0.0011	0.0013	0.0014
	1–5 year olds	0.0016	0.0020	0.0023	0.0026
	Mean across all other age groups	0.0031	0.0038	0.0044	0.0050
Q* from Approach 1 (95% CI, ls^**-1**^**)**		1977 (1341–2614)	2407 (1632–3181)	2810 (1906–3714)	3190 (2163–4216)
Q* from Approach 2 (95% CI, ls^**-1**^**)**		2258 (1629–3704)	2743 (2139–4429)	3200 (2470–5346)	3639 (2765–5767)

*CI: confidence interval; CO_2_: carbon dioxide; MET: metabolic equivalents; Q: absolute ventilation rate.

Regardless of the approach used, the resulting estimates of the absolute ventilation rate, Q, increased by approximately 400 ls^-1^ for each 0.2 MET increase in the assumed metabolic activity level.

Differences were evident in the data between the first and last five observations (Fig 1 and Table 2), and we therefore estimated ventilation rates separately for the two time periods. Estimates of Q were similar using the first five observation compared to using all ten observations (2510 ls^-1^ compared to 2407 ls^-1^ using approach 1, and 2571 ls^-1^ compared to 2743 ls^-1^ using approach 2.). Neither approach gave meaningful results using the last five observations only (see S1 File).

Finally, we showed that our results are not overly sensitivity to greater gaps in time between observations (see S1 File).

## Discussion

The role of airborne transmission of SARS-CoV-2 in the COVID-19 pandemic has brought to the forefront the critical need for adequate ventilation in indoor congregate settings such as clinic waiting rooms. Improved ventilation would not only potentially reduce COVID-19 deaths, but would also reduce the high numbers of deaths that continue to occur from other airborne infectious diseases such as tuberculosis [6]. It can be difficult to estimate ventilation in these settings however, and the approaches that are typically used in epidemiology do not account for fluctuating occupancy and CO_2_ concentration over the course of a day. In this paper, we demonstrate a simple method that overcomes these limitations, and is suitable for widespread use both in epidemiological research and by facility managers.

To help prevent transmission of pathogens by the airborne route, the World Health Organization has previously recommended natural ventilation of at least 60 ls^-1^/patient for general outpatient departments and wards [15]. In this study, the average absolute ventilation rate of the clinic waiting room was estimated to be 120 ls^-1^/patient using approach 1 and 137 ls^-1^/patient using approach 2.

Both non-steady state approaches produced similar estimates of the absolute ventilation rate with a relative difference in Q of 13% between the two approaches. However, approach 1 did not require room volume measurements and was technically and computationally less intensive than approach 2. Approach 1 produces estimates that are likely to be sufficiently accurate for most applications, and the analyses are considerably simpler to conduct. However, it is worth noting that approach 2 may work better in poorly ventilated spaces where CO2 levels may take some time reach equilibrium, as the method does not assume equilibrium is instantaneously achieved. Both approaches need further validation.

The estimated 95% confidence intervals were large, with a range of 1549 with approach 1, and 2290 with approach 2. These confidence intervals should be interpreted as reflecting both the uncertainty we have in the true ventilation rate, but also any variation in the ventilation rate that occurred over the 3 hour data collection period. For instance, due to windows being open or closed, or changes in wind speeds or direction.

We only present results for one space in a single clinic, recorded on one day only. As such, our results are not designed to be representative of clinics in the province, or even of the clinic as a whole. When applying these methods elsewhere, there are a number of adaptations to the data collection method described here that could improve the accuracy and generalisability of ventilation estimates. Firstly, the duration of data collection was only 3 hours 10 mins for the dataset used in this study. The outputs may, therefore, not be representative of a full clinic day. Specifically, the time with the highest occupant density (early mornings) was not captured. Additionally, there is likely to be substantial variation in ventilation rates between days, as a result of differences in daily wind speed, wind direction, and whether doors and windows were opened or closed, and more generally, seasonality. Using data collected over a range of days and weather conditions would help produce a more accurate and representative estimate of absolute ventilation. Taking more regular CO_2_ measurements over a longer period can be easily done, particularly if meters can be left in situ [13]. Recording CO_2_ measurements and headcount data at more frequent intervals may also improve estimates, although our sensitivity analysis suggests that the method is not overly sensitive to moderate gaps between observations (S1 File).

All occupants were assumed to have the same level of metabolic activity (although the variation in CO2 generation rates between age groups was taken into account). Sensitivity analyses showed that a slight change in assumed activity levels (such as sitting quietly [1.0–1.3 MET] vs sitting with light tasks such as doing office work [1.5 MET]) resulted, in this space, in an increase of approximately 500 ls^-1^ in the estimated absolute ventilation rate per 0.2 MET change in activity. A better understanding of the metabolic rate of individuals in clinical and other congregate spaces could help resolve this uncertainty. Both approaches assume that air is well mixed. The three indoor CO_2_ dataloggers, situated in different places in the room, recorded very similar values to each other for most of the data collection period, suggesting that this assumption was reasonable. Their values differed from each other at the start of the period, however, and the assumption may therefore not have been true for the first part of the data collection. Additionally, both approaches assume that the replacement air comes only from the outside space where the meter is located. Where spaces adjoin other occupied spaces and exhaled breath from adjacent spaces make a contribution to CO_2_ levels, the absolute ventilation rate may be underestimated. However, ventilation from other occupied areas will likely not result in the same reductions in transmission risk, and so this is not a major limitation.

Finally, Table 2 and Fig 1 show a notable difference between data in the first half of the morning, where attendance was high and the indoor CO_2_ concentration was well above the outdoor concentration, and data in the second half of the morning, where few people were in the room and the levels of indoor and outdoor CO_2_ were very similar. For this reason, we used approaches 1 and 2 to estimate the absolute ventilation rate in the first half/second half of the morning separately (cf. S1 File for more details on this analysis). While both approaches worked well on the first five observations, they led to unreliable estimates for Q when applied to the last five observations. This is likely due to the difference between outdoor and indoor CO_2_ concentration being below the accuracy of the instrument for all last five observations. This demonstrates that these methods may fail in settings were numbers of people are low and ventilation rates high, although this could be mitigated by the use of more precise CO_2_ dataloggers. As government-mandated lockdowns are lifted in many countries, and people return to crowded congregate settings, a simple and readily scalable method may help to identify spaces where inadequate ventilation may result in high SARS-CoV-2 transmission risk. Methods that calculate the absolute ventilation rate are preferable, as approaches that calculate only transmission risk fail to partition that risk into overcrowding versus inadequate ventilation–problems with distinct solutions.

The method demonstrated in this study improves on the existing approaches typically used in epidemiological research, by allowing ongoing estimation of ventilation levels in busy spaces where the number of people present and ventilation rate may change over time. Data collection requires only a CO_2_ meter and minimal training. The proposed analysis could be readily programmed into a mobile phone application or an online calculator. In summary, of the two approaches explored in this paper, we would recommend approach 1 and suggest further work to validate the method in other settings. This could include comparing the CO_2_ release method with the approaches used in this study, or taking simultaneous measurements with balometers. However, we note that such comparisons are inherently limited. The former by the fact that contemporaneous measurement is not possible, given one approach requires to space to be occupied, and the other requires it to be empty. The latter as balometers could not be used on all ventilation points in a space in which people are entering and exiting.

Simple reorganisation of the workplace or low cost retrofits can have a significant impact on the absolute ventilation rate [12–14, 23]. Empowering clinicians, facility managers and disease intervention programmes to identify inadequately ventilated spaces is a necessary first step in reducing the risk of acquiring airborne infectious diseases in congregate settings such as healthcare facilities.

## Supporting information

S1 File Contains all of the S1 and S2 Tables. (DOCX)
