# Supplementary material for: Estimating ventilation rates in rooms with varying occupancy levels: Relevance for reducing transmission risk of airborne pathogens
Source: PLoS One. 2021 Jun 24;16(6):e0253096. doi: 10.1371/journal.pone.0253096 (PMC8224849; doi:10.1371/journal.pone.0253096)
Supplement: S1 File — (DOCX) [file pone.0253096.s001.docx]

# **Supporting information**

Separate analysis of first and last five observations

From Table 2 and Fig 1 it is evident that there is a large difference between values in the first half and the second half of the collected data. In particular, while the first five observations show a high number of occupants and a larger difference between outdoor and indoor CO_2_ levels, the last five observations indicate low numbers of occupants and little variation between indoor and outdoor CO_2_ levels. To take this dissimilarity into consideration, we analysed the first half and the second half of the data separately, using both approaches applied in this article.

Table S1 shows the results of applying both approaches to the first five observations. The two methods give very similar estimates of Q and SSR. Furthermore, the Q estimates we get here are compatible with the values of Q found using all collected data (Table 3).

**S1 Table. Results of approach 1 (linear regression) and approach 2 (model fit) for estimating the absolute ventilation rate (Q) in the clinic waiting room during time period 9:40-11.03**

|  | **Approach 1** | **Approach 2** |
| --- | --- | --- |
| **Room use** | Waiting area | |
| **Volume of space (l)** | 135363 | |
| **Duration of measurement (s)** | 4980 | |
| **SSR** | 9.3 x 10^-10^ | 7.4 x 10^-10^ |
| **Absolute ventilation rate, Q (95% CI, l/s)** | 2510 (1495 - 3524) | 2571 (2079- 4098) |

In the analysis of the second half of the collected data, approach 1 produced a negative estimate of Q (-2575 l/s), while approach 2 did not converge to a finite value, suggesting very high estimates of Q. It is clear that results from both approaches are unreliable in this case.

# **Supporting information**

Sensitivity of ventilation rate estimates to removal of observations

To evaluate the error sensitivity of the numerical methods used to approximate equation 9, we evaluate the sensitivity of the ventilation rate estimate to greater gaps between the CO2 observations, by removing one observation at a time (out of the ten available). This provides a crude indication of the sensitivity of the methods to the non-continuous nature of the data, and therefore the sensitivity of the numerical methods.

For each of the 10 data subsets, Table S2 shows the estimates of ventilation rates we obtained from approach 1 and approach 2. The majority of estimates are within ±10% of the main estimates, however removal of the 2^nd^ observation results in estimates that are 18% and 29% higher the main estimates (approach 1 and 2 respectively). These are still well within the 95% confidence intervals for the main estimates however.

**S2 Table. Results of approach 1 (linear regression) and approach 2 (model fit) for estimating the absolute ventilation rate (Q) using only nine out of the ten available observations.**

| **Observation removed** | **1^st^** | **2^nd^** | **3^rd^** | **4^th^** | **5^th^** | **None** |
| --- | --- | --- | --- | --- | --- | --- |
| **Q estimate and 95% CI Approach 1** | 2208  [13403077] | 2838  [1833-3842] | 2412  [1476-3348] | 2264  [1466-3062] | 2351  [1554-3148] | 2407  [1632-3181] |
| **Q estimate and 95% CI Approach 2** | 2732  [2197-4770] | 3547  [2672-5280] | 2879  [2113-5231] | 2488  [2039-4040] | 2606  [2075-4455] | 2743  [2139-4429] |

| **Observation**  **removed** | **6^th^** | **7^th^** | **8^th^** | **9^th^** | **10^th^** | **None** |
| --- | --- | --- | --- | --- | --- | --- |
| **Q estimate and 95% CI Approach 1** | 2455  [1723-3186] | 2438  [1666-3211] | 2427  [1625-3229] | 2409  [1575-3243] | 2406  [1570-3242] | 2407  [1632-3181] |
| **Q estimate and 95% CI Approach 2** | 2655  [2047-4436] | 2691  [2119-4577] | 2717  [2131-4672] | 2741  [2123-4665] | 2741  [2132-4651] | 2743  [2139-4429] |
